# The Growth and Decay of Intense GNSS Amplitude and Phase Scintillation During Non‐Storm Conditions

**DOI:** 10.1029/2024SW004108

**Published:** 2024-11-30

**Authors:** Mahith Madhanakumar, Andres Spicher, Juha Vierinen, Kjellmar Oksavik, Anthea J. Coster, Devin Ray Huyghebaert, Carley J. Martin, Ingemar Häggström, Larry J. Paxton

**Affiliations:** ^1^ Department of Physics and Technology UiT The Arctic University of Norway Tromsø Norway; ^2^ Department of Physics and Technology University of Bergen Bergen Norway; ^3^ Arctic Geophysics University Centre in Svalbard Longyearbyen Norway; ^4^ Haystack Observatory Massachusetts Institute of Technology Westford MA USA; ^5^ Institute of Space and Atmospheric Studies University of Saskatchewan Saskatoon SK Canada; ^6^ EISCAT Scientific Association Kiruna Sweden; ^7^ The Johns Hopkins University Applied Physics Laboratory Laurel MD USA

**Keywords:** amplitude scintillation, phase scintillation, GNSS, polar ionosphere, IMF reversals

## Abstract

A multi‐instrument study is conducted at the dayside polar ionosphere to investigate the spatio‐temporal evolution of scintillation in Global Navigation Satellite System (GNSS) signals during non‐storm conditions. Bursts of intense amplitude and phase scintillation started to occur at ∼ 9 MLT and persisted for more than 1 hour implying the simultaneous existence of Fresnel and large‐scale sized irregularities of significant strength in the pre‐noon sector. Measurements from the EISCAT radar in Svalbard (ESR) revealed the presence of dense plasma structures with significant gradients in regions of strong Joule heating/fast flows and soft precipitation when scintillation was enhanced. Plasma structuring down to Fresnel scales were observed both in the auroral oval as well as inside the polar cap with the associated amplitude scintillation exhibiting similar strengths regardless of whether the density structures were in regions of active auroral dynamics or not. The observations are placed within the context of different sources of free energy, providing insights into the important mechanisms that generate irregularities capable of perturbing GNSS signal properties in the dayside ionosphere. Furthermore, a strong negative excursion in the interplanetary magnetic field (IMF) $B_{y}$ component during the northward turning of $B_{z}$ led to the transport of a depleted region of plasma density into the post‐noon sector that significantly weakened both amplitude and phase scintillation.

## Key Points

- Intense amplitude and phase scintillation in the pre‐noon sector during non‐storm conditions
- Structuring down to Fresnel scales in both the auroral oval and inside the polar cap
- Depletion of Fresnel structures due to IMF *B*_*z*_ and *B*_*y*_ reversal in the post‐noon sector

## 1 Introduction

Global Navigation Satellite Systems (GNSS) have become an integral part of our modern society with its services such as positioning, navigation and timing (PNT) finding application in a variety of fields ranging from personal navigation to agriculture, surveying, environmental modeling and aviation. Interruptions in GNSS services can arise when the signal properties are modified by plasma irregularities as the signal traverses the ionosphere (Kintner et al., 2001, 2007). Electron density irregularities frequently form in large‐scale plasma structures with significant electron density gradients, leading to positioning errors that can even exceed 10 m at high latitudes during severe geomagnetic conditions (Yang et al., 2020). In addition to range errors, irregularities can also cause rapid fluctuations in the amplitude and phase of L‐band signals, which is commonly referred to as scintillation (Yeh & Liu, 1982). Scintillation in the amplitude of a signal is due to diffraction of radio signals by Fresnel sized‐irregularities, which are of the order of ∼350 m at 350 km for the GPS $L_{1}$ frequency. On the other hand, perturbations in the signal phase can arise from diffractive fluctuations induced by Fresnel scaled irregularities (i.e., scintillation) and refractive fluctuations induced by large‐scale irregularities convecting across the signal path with increased drift velocities at high latitudes (Kintner et al., 2007). In the literature, perturbations in phase due to both diffraction and refraction are collectively referred to as phase scintillation (Kintner et al., 2007). This paper will use the term Fresnel scaled irregularities to refer to small‐scale irregularities that cause diffractive scintillation and large‐scale irregularities to refer to density structures that predominantly causes refraction in GNSS signals. Even though refractive fluctuations from the phase can be corrected by using the ionosphere‐free linear combination (IFLC), the technique fails to remove the ionospheric contribution during scintillation when diffractive contributions are simultaneously present (Carrano et al., 2013; McCaffrey & Jayachandran, 2019; Zheng et al., 2022). From the perspective of space weather impacts on GNSS technologies, both phase scintillation (diffractive + refractive) as well as amplitude scintillation are equally important. Intense phase scintillation can cause loss‐of‐lock in signals. On the other hand, simultaneous amplitude and phase scintillation can also result in loss‐of‐lock when tracking the phase plus deep amplitude fadings below the receiver threshold for continuous signal tracking, thereby impairing the technologies using GNSS receivers (Jiao & Morton, 2015; Jin & Oksavik, 2018; Liu et al., 2017; Semeter et al., 2017).

Scintillation studies at high latitudes have primarily reported phase scintillation in relation to polar cap patches, auroral structures, particle precipitation events and field‐aligned currents (Alfonsi et al., 2011; Faehn Follestad et al., 2020; Forte et al., 2017; Jin et al., 2014, 2017; J. I. Moen et al., 2013; Oksavik et al., 2015; Smith et al., 2008; Prikryl et al., 2011; van der Meeren et al., 2015). Even though phase scintillation occurs more predominantly than amplitude scintillation at high latitudes, there have been some studies that focused on the simultaneous occurrence of amplitude and phase scintillation events. For instance, using the total electron content (TEC) and scintillation data collected during the Halloween storms of 2003, De Franceschi et al. (2008), Mitchell et al. (2005) showed amplitude and phase scintillation to be co‐located in regions of large TEC gradients in the nightside sector. The climatology study over northern Europe by Spogli et al. (2009) revealed the enhancement of $S_{4}$ index at the noon and midnight sectors under both quiet and disturbed geomagnetic conditions. Using GPS $L_{1}$ data from the Canadian High Arctic Ionospheric Network (CHAIN), Meziane et al. (2020) investigated the seasonal and solar cycle dependence of amplitude scintillation during the 24th solar cycle revealing a maximum occurrence value of ∼11% during the early winter periods of high solar activity. Thayyil et al. (2021) reported significant amplitude and phase scintillation in relation to a polar cap patch at the nightside sector where non‐linear evolution of the gradient drift instability mechanism was suggested to be responsible for generating Fresnel scale structures throughout the patch. Using observations from Skibotn in northern Norway, Jin and Oksavik (2018) studied strong amplitude and phase scintillation together with power fading and signal loss‐of‐locks associated with TEC blobs during the severe storm on St.Patrick's Day in 2015. A multi‐instrument approach to investigating the favorable conditions triggering amplitude scintillation in the southern hemisphere during the main phase of a severe geomagnetic storm in September 2017 was conducted by D’Angelo et al. (2021). They suggested high ionization levels and strong plasma dynamics as the necessary conditions for amplitude scintillation occurrence. Using scintillation indices at 1‐s resolution, Nishimura et al. (2023) associated amplitude and phase scintillation with different auroral forms at the nightside sector during a substorm. Cardenas‐O’Toole et al. (2024) observed moderate levels of amplitude and phase scintillation at the dayside convection reversal boundary and which coincided with regions of large flow shear and enhanced soft electron precipitation.

As mentioned above, a large fraction of studies observed amplitude scintillation during periods of severe geomagnetic disturbances in the noon or nightside sectors of the high latitude ionosphere. Less attention has been paid to understanding the ionospheric effects on GNSS signals during weak or quite geomagnetic periods (i.e., when SYMH ≥−80 nT following the definition of a weak storm in Hutchinson et al. (2011)). In this study, we present evidence for the occurrence of intense amplitude and phase scintillation in the pre‐noon sector along with a significant reduction of scintillation in the post‐noon sector during non‐storm conditions on November 2013. The analysis was carried out using co‐ordinated observations from a network of both ground and space based instruments that revealed the spatio‐temporal evolution of irregularities of different scale‐sizes and the associated scintillation in GNSS signals at the dayside high latitude ionosphere.

The outline of the paper is as follows. In Section 2, we provide an overview of the data and different instruments used in the study. In Section 3, the methodology employed to combine observations from multiple instruments is discussed followed by the results in Section 4. Discussion of the results are offered in Section 5 with the key points summarized in the conclusions of Section 6.

## 2 Data and Instrumentation

Being a multi‐instrument study we make use of a number of space‐borne and ground‐based instruments to characterize scintillation in GNSS signals at the high latitude ionosphere. An overview of the instruments (or data sources) used in the study is given below with a quick summary given in Table A1.

### 2.1 EISCAT Svalbard Radar (ESR)

Ionospheric parameters such as electron density $\left(N_{e}\right)$, electron temperature $\left(T_{e}\right)$, ion temperature $\left(T_{i}\right)$ and line‐of‐sight plasma velocity $\left(V_{i}\right)$ are collected from a wide range of latitudes by the steerable 32‐m ESR dish operated in a scanning mode with a minimum elevation of 30° above the horizon (Wannberg et al., 1997). As the radar was simultaneously moving in azimuth, data from different longitude sectors are also available in this mode. Each scan is completed in about 12 minutes yielding *fan plots* of $N_{e}$, $T_{e}$, $T_{i}$ and $V_{i}$ as a function of altitude and latitude, produced with an integration time of ∼30s (Carlson et al., 2002). Data with errors greater than 40% are discarded from the analysis. Additionally, the measurements from the co‐located field‐aligned ESR‐42 m, produced with an integration time of ∼60s, are used to complement the observations from ESR‐32 m. The ESR measurements are analyzed using the GUISDAP toolbox (Lehtinen & Huuskonen, 1996).

### 2.2 GNSS Ionospheric Scintillation and TEC Monitors (GISTM)

Scintillation and TEC data primarily used in this study are collected by a NovAtel GPStation‐6 GNSS Ionospheric Scintillation and TEC Monitor (GISTM) installed at the Kjell‐Henrikson Observatory (KHO) in Longyearbyen. This receiver is capable of simultaneously tracking signals at several different frequencies and from different constellations including GPS, GALILEO and GLONASS (Oksavik, 2020a). Amplitude and phase scintillation indices $\left(S_{4},\sigma_{\phi }\right)$ together with TEC and ROT data with 60‐s resolution are the primary data sets used from this instrument. These 60‐s resolution indices are calculated and recorded automatically by the receiver. The $S_{4}$ index is calculated from raw amplitude whereas a sixth‐order Butterworth high‐pass filter is used to detrend raw phase and calculate the $\sigma_{\phi }$ index (Fremouw et al., 1978; Van Dierendonck et al., 1993). Vertical TEC is obtained from slant TEC assuming an ionospheric pierce point (IPP) at 350 km (Horvath & Crozier, 2007). Additionally, raw carrier phase and amplitude, recorded at a sampling rate of 50 Hz, were used to re‐calculate $S_{4}$ and $\sigma_{\phi }$ indices at 1‐s resolution. Using the 1‐s window would allow us to better capture the intense and short‐lived bursts in scintillation that otherwise would be averaged out if using the traditional 1‐min window. Similar to the 60‐s resolution indices, the 1‐s $S_{4}$ was calculated from raw power whereas $\sigma_{\phi }$ at 1‐s resolution was obtained from the detrended phase. Only data corresponding to elevation angles greater than 40° are considered to avoid multipath effects. More information about data processing can be found in Oksavik et al. (2015) and van der Meeren et al. (2015). When presenting TEC maps, scintillation and TEC data collected by the receivers in Bjørnøya (BJN) and Ny‐Ålesund (NYA) are additionally used to expand the data coverage. These data set are part of The University of Bergen GNSS Data Collection (Oksavik, 2020b).

### 2.3 Madrigal Total Electron Content (TEC)

The plasma variability at global scales are captured using the GPS vertical TEC data from the Madrigal database (Coster, 2013). Bins of 1°×1° in geographical latitude‐longitude, generated every 5 min, contain VTEC values that are calculated from the line‐of‐sight TEC assuming an ionospheric slab at 350 km altitude (Rideout & Coster, 2006). Methods implemented to compute GPS receiver biases for TEC measurements in the Madrigal database are described in Vierinen et al. (2016). In this study we use data from latitude ranges of 60°–90° to generate TEC maps in Magnetic Local Time (MLT) and Altitude‐Adjusted Corrected Geomagnetic (AACGM‐v2) coordinates. The readers are referred to Shepherd (2014) and Burrell et al. (2020) for more information on AACGM‐v2 co‐ordinates as well as for converting between geographic and geomagnetic co‐ordinates.

### 2.4 Super Dual Auroral Radar Network (SuperDARN)

SuperDARN is a network of globally distributed High Frequency (HF) coherent scatter radars used to image large‐scale ionospheric convection and plasma dynamics in the polar, auroral, sub‐auroral and mid‐latitude regions (Chisham et al., 2007; Nishitani et al., 2019). Two dimensional **E** × **B** velocity of the high latitude plasma is measured by observing the drift of field‐aligned irregularities in the F region (Ruohoniemi & Baker, 1998). Data from the northern hemisphere radars are used to generate maps of velocity and electric potential using the SuperDARN community “Radar Software Toolkit” (SuperDARN Data Analysis Working Group, 2022). In this study we only use data covering a latitude range of 60°−90° in the MLT ‐ AACGM‐v2 coordinates (Burrell et al., 2020; Shepherd, 2014; SuperDARN Data Visualization Working Group, 2024). Together with the TEC maps, use of SuperDARN data allows association of the global evolution of the ionospheric flow to the movement of large density structures in the polar ionosphere.

### 2.5 Special Sensor Ultraviolet Spectrographic Imager (SSUSI)

SSUSI is a far ultraviolet (UV) instrument consisting of a Spectrographic Imaging System (SIS) mounted on a nadir‐looking panel of the Defense Meterological Satellite Program (DMSP) satellites F16‐F19, that are in a nearly polar, sun‐synchronous orbits at an altitude of about 850 km. SSUSI records UV emissions from the Earth's upper atmosphere in five wavelength bands in the spectral range 115–180 nm by scanning across the track of the DMSP trajectory every 15 s (Paxton et al., 1992, 2002). In this study, we make use of the poleward and equatorward auroral boundary locations obtained from the Auroral Environmental Data Record (EDR).

### 2.6 OMNI

In order to study the impact of Interplanetary Magnetic Field (IMF) on the ionospheric dynamics and hence on GNSS scintillation we make use of the 1‐min high resolution OMNI data set timeshifted to the nose of Earth's bow shock (King & Papitashvili, 2005). Of particular relevance to this study are the IMF $B_{y},B_{z}$ components in Geocentric Solar Magnetospheric (GSM) coordinates, solar wind flow speed and pressure. Additionally, as a proxy for the geomagnetic storm strength, the 1‐min resolution SYMH index that captures the disturbances in the symmetric horizontal component, H, is used (World Data Center for Geomagnetism, Kyoto, 2022). The Auroral Electrojet (AE), Auroral Upper (AU) and Auroral Lower (AL) indices with 1‐min resolution are used to capture the ionospheric disturbances at high latitudes (World Data Center for Geomagnetism, Kyoto, 2015).

## 3 Methodology

In this section, we briefly outline the procedure developed to combine and present the data sets from different sources. The $S_{4}$ and $\sigma_{\phi }$ of a given PRN were calculated from all the available frequencies. In this study, we used the maximum value of the $S_{4}$ and $\sigma_{\phi }$ to capture the maximum level of scintillation at any given time period. $S_{4}$ values are corrected for noise by using the equation (Van Dierendonck et al., 1993):

(1) $$S_{4}=\sqrt{S_{\mathrm{4tot}}^{2}-S_{\mathrm{4cor}}^{2}}$$

where $S_{\mathrm{4tot}}$ is the total $S_{4}$ that include contributions from both ionosphere and the ambient noise whereas $S_{\mathrm{4cor}}$ is the correction to total $S_{4}$ due to the effect of ambient noise. An event is classified as amplitude scintillation if $S_{4}\geq 0.1$ and phase scintillation if $\sigma_{\phi }\geq 0.1$ rad. Due to Fresnel filtering effect, the dominant contribution to amplitude scintillation comes from irregularities with scale‐sizes of the order of couple of hundreds of meters or less whereas irregularities with scale‐sizes ranging from several meters to kilometers can contribute to phase scintillation (Forte & Radicella, 2002; Yeh & Liu, 1982).

All TEC maps are overplotted with $S_{4}$ values whereas SuperDARN convection maps contain $\sigma_{\phi }$ values (see Figures 2, 3, 4). In addition, the zoomed versions of the TEC maps also contain the look directions of the scanning ESR‐32 m beams as gray dotted lines and the field‐aligned ESR‐42 m beams as purple lines. Since the TEC maps are of 5 min resolution and the scintillation indices are of 1‐min resolution, the maximum value of the scintillation indices during every 5 min interval, for each PRN, are only overplotted for clarity and to avoid cluttering. Since the SuperDARN maps are of 2 min resolution, the average of the convection pattern and velocities during each 5 min interval is taken. In addition, the convection maps also show the poleward and equatorward edges of the auroral boundary obtained from DMSP SSUSI. If there are data from multiple DMSP satellites corresponding to any TEC timestamp, the average of the boundary locations are taken. Furthermore, in cases when there are no data, we use the boundary locations corresponding to the nearest TEC timestamp when data was available. The IMF $B_{y},B_{z}$ clock plots are also shown alongside the TEC maps.

When presenting the ESR‐32 m fan plots (see Figure 5), $S_{4}$ values are always overplotted on top of $N_{e}$ and $T_{e}$ scans whereas $T_{i}$ and $V_{i}$ plots contain the $\sigma_{\phi }$ values. Conjunctions between GNSS signals and scanning radar beams are identified using the procedure outlined in Madhanakumar et al. (2024). In our study, a conjunction is defined to occur if a PRN link falls within a (lat, lon) threshold of (±0.5°,±2°), at an IPP of 350 km, of a radar beam. A similar procedure is used to identify conjunctions between the field‐aligned ESR‐42 m and GNSS signals. By integrating the densities observed by the ESR‐42 m along each beam, the corresponding TEC values are calculated which are then converted to vertical TEC $\left(VTEC^{\mathrm{radar}}\right)$. In addition, the gradients in density are obtained using the following equation:

(2) $$\nabla N_{e}=\frac{\Delta N_{e}}{\Delta x}$$

where Δx=v×Δt. Here, $\Delta N_{e}=N_{e}\left(t_{i+1}\right)-N_{e}\left(t_{i}\right)$, $\Delta t=t_{i+1}-t_{i}$, v = average velocity of plasma flow in a 2 × 2 grid of (lat, lon) around the ESR‐42 m location obtained from SuperDARN. Note that $\nabla N_{e}$ corresponds to the component of density gradient along the **E** × **B** drift direction.

## 4 Results

Having given a brief overview of the methodology, we now proceed to present the results of the multi‐instrument study aimed to characterize intense levels of amplitude and phase scintillation during non‐storm conditions. We first present the prevailing IMF conditions and the corresponding scintillation observations from KHO. Subsequently, we show the behavior of the ionosphere at global scales and its response to the IMF $B_{y},B_{z}$ components. Finally, we show the persisting ionospheric conditions above Svalbard during different intervals of scintillation.

### 4.1 IMF and Scintillation/TEC Behavior

**Figure 1:**
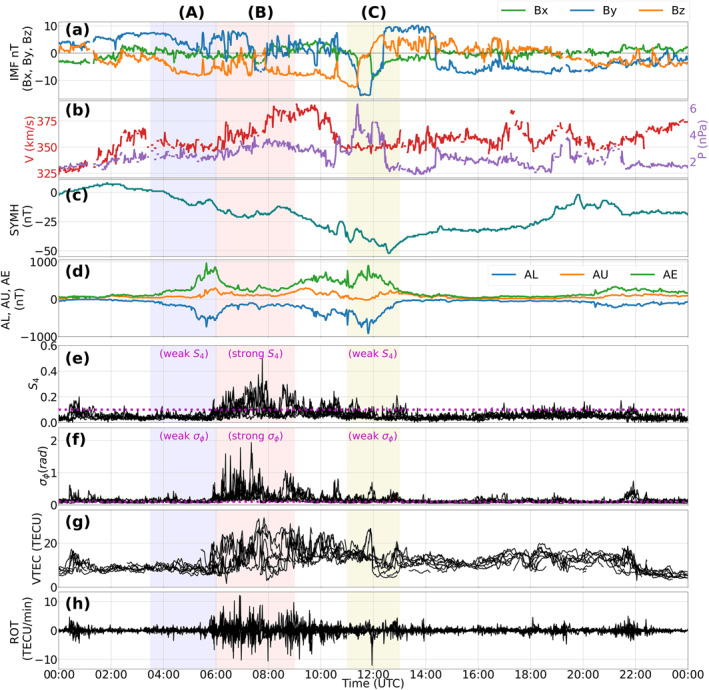
Interplanetary magnetic field conditions (panels a–b), SYMH index (panel c), auroral electrojet indices (panel d), $S_{4},\sigma_{\phi }$ indices (panels e–f), GNSS VTEC and ROT (panels g–h) on 07 November 2013. In panels (e–h), data from all available PRNs have been included and a magenta dotted horizontal line in panels (e–f) show the threshold of 0.1 used to separate scintillation events from non‐scintillation ones. The average scintillation intensities during the three intervals, (A), (B), (C), have been categorized as “weak” or “strong” to facilitate easy comparison of scintillation strengths across intervals.

Panels (a–d) of Figure 1 presents the IMF conditions during the $7^{th}$ of November 2013 together with the SYMH and AE indices. Figure 1c shows negative excursions in the SYMH index implying some enhancements in the ring current activity. However, as discussed in Gonzalez et al. (1994), every ring current enhancement cannot be considered as a signature of storm level disturbances. Hutchinson et al. (2011) classified a storm as weak if −150<SYMH≤−80 nT. Since the minimum value of SYMH index observed in our study was only −52 nT, our event does not even fall within the definition of a weak storm. We therefore consider our event as a typical example of a non‐storm event. The 1‐min resolution scintillation indices, $S_{4}$ and $\sigma_{\phi }$, from all the satellites in the field‐of‐view during the given day are shown on panels (e–f) whereas the corresponding VTEC and ROT values are on panels (g–h). Three intervals separated in time were selected to illustrate the spatio‐temporal evolution of the scintillation strength, beginning with a state of low activity followed by a period of intensification and a final reduction to background levels. These intervals are labeled (A), (B) and (C) respectively and are shaded in different colors to aid visual clarity (see Figure 1). Interval (A) corresponds to the time period between 03:30–06:00 UTC (∼ 06:30–09:00 MLT), interval (B) between 06:00–09:00 UTC (∼ 09:00–12:00 MLT) and interval (C) between 11:00–13:00 UTC (∼ 14:00–16:00 MLT). Relative to the average cusp location, interval A is therefore in the morning sector, interval B is distributed between the prenoon and noon sectors whereas interval C is in the afternoon sector.

**Figure B1:**
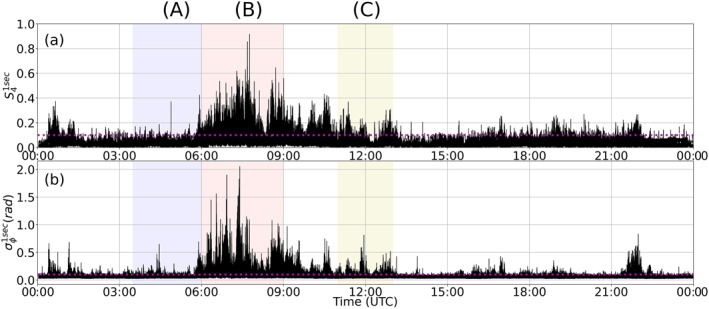
$S_{4}$ (panel a), $\sigma_{\phi }$ (panel b) indices with a resolution of 1‐s on 07 November 2013. Data from all available PRNs of different GNSS constellations have been included. A magenta dotted horizontal line, in both panels, show the threshold of 0.1 used to distinguish between scintillation and non‐scintillation events.

During interval (A), IMF $B_{z}$ was negative reaching a minimum value of −8 nT at 05:20 UTC. The SYMH index starts decreasing in this interval after a prior increase. The AE index increases and reaches a maximum of ∼1000 nT indicating substorm activity. The $S_{4}$ index remained below the threshold of 0.1 majority of the times whereas $\sigma_{\phi }$ fluctuated between 0.05 and 0.2 rad on average (see also Figure B1).

In interval (B), IMF $B_{z}$ remained southward majority of the times even though there were some small northward excursions. On the other hand, though the $B_{y}$ component exhibited some negative turnings reaching a minimum value of about −7 nT at 07:40 UTC, it remained mostly positive. The solar wind velocity is seen to increase during this interval reaching a maximum value of 390 km/s whereas the SYMH index barely exceeded −20 nT. The AE index decreased and remained below 450 nT majority of the time. It is during this interval when intense levels of amplitude and phase scintillation were observed. As seen in panels (e–f), both $S_{4}$ and $\sigma_{\phi }$ simultaneously experienced bursts in scintillation that lasted for ∼ 1 hr reaching a maximum value of 0.5 and 1.9 rad respectively. The VTEC and ROT panels also showed significant enhancements from the background with the VTEC reaching a maximum value of 30 TECU whereas ROT maximized at 12 TECU/min. In addition, as shown in Figure B1 of the appendix, the 1‐s amplitude and phase scintillation indices showed larger enhancements than the 1‐min indices with $S_{4}$ and $\sigma_{\phi }$ reaching a maximum value of 0.75, 2.2 rad respectively confirming that interval (B) was the most intense period of scintillation during the day.

Finally, interval (C) corresponds to the time period when IMF $B_{z}$ turned positive after reaching a minimum value of −13 nT at 11:15 UT and thereafter staying positive or around zero until 19:00 UT after which it further starts decreasing. $B_{y}$ was strongly negative during this interval reaching a minimum of −15.5 nT at 11:30 UT beyond which it flipped back to positive values of about 10 nT at 12:20 UT. This interval is associated with a region of enhanced solar wind pressure and a reduction in the solar wind velocity with the SYMH index reaching the minimum value of −52 nT. The AE index was again enhanced reaching a maximum value of 900 nT indicating ongoing substorm activity in the nightside ionosphere. The $S_{4}$ and $\sigma_{\phi }$ strengths are observed to fade out during this interval with $S_{4}<0.1$ on majority of the GNSS links whereas $\sigma_{\phi }$ remained above the threshold though with reduced intensity as compared to interval (B). Clear differences in the magnitudes of VTEC and ROT are also seen in comparison to interval (B).

### 4.2 Global Behavior

**Figure 2:**
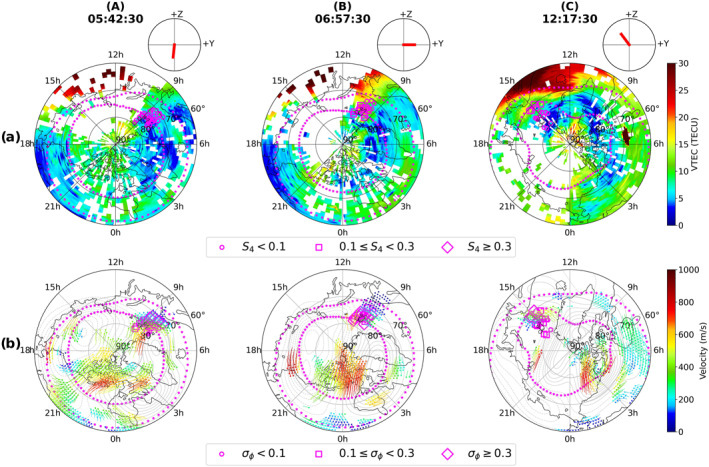
Panel (a): TEC maps corresponding to times within the same three intervals (A), (B), (C) as in Figure 1. Panel (b): SuperDARN convection maps along with poleward and equatorward auroral boundaries. $S_{4}$ indices are overplotted on the TEC maps whereas $\sigma_{\phi }$ values are overlaid on the SuperDARN maps. The interplanetary magnetic field clock plots are shown for each timestamps above the TEC maps.

**Figure 3:**
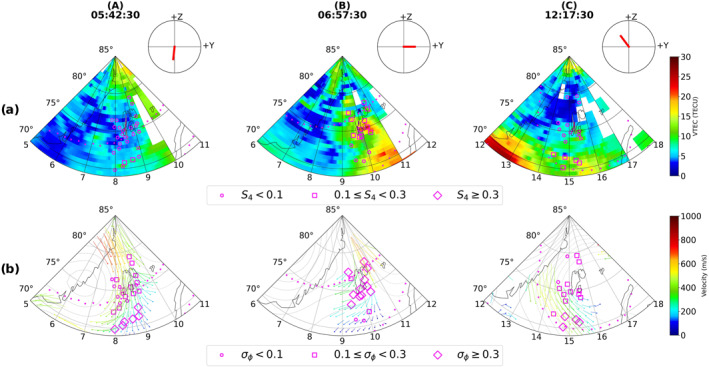
Zoomed in version of the TEC and SuparDARN maps above Svalbard with scintillation indices and DMSP SSUSI auroral boundaries overplotted. The interplanetary magnetic field clock plots are shown for each timestamps above the TEC maps.

In order to investigate the global ionospheric behavior during the aforementioned three intervals, we make use of the global TEC and SuperDARN convection maps. These maps are shown in Figure 2 which also contain the IMF clock plots as well as the poleward and equatorward auroral boundaries from DMSP SSUSI. In addition, the $S_{4}$ and $\sigma_{\phi }$ values are overplotted on top of the TEC and SuperDARN maps respectively. As mentioned earlier, for a given time and PRN, the maximum of $S_{4}$ and $\sigma_{\phi }$ values from the available frequencies are used. A zoomed in version of the TEC and SuperDARN maps above Svalbard archipelago, in the same format as Figure 2, is shown in Figure 3.

During interval A, $B_{y}$ is mostly positive and hence the cusp is shifted toward later MLT bringing the interval further into the early morning local time. As seen in Figure 2b, enhanced regions of flow were visible in the dawn sector in the vicinity of the GNSS signals. Weak‐moderate $\sigma_{\phi }$ was observed on GNSS links whereas $S_{4}$ remained below 0.1 majority of the times. As seen in Figure 3b, moderate $\sigma_{\phi }$ occurred near the inflow region and inside the auroral oval whereas $\sigma_{\phi }$ remained weak poleward and inside the polar cap. The corresponding TEC values in the vicinity remained low and varied between 5 and 10 TECU.

**Figure 4:**
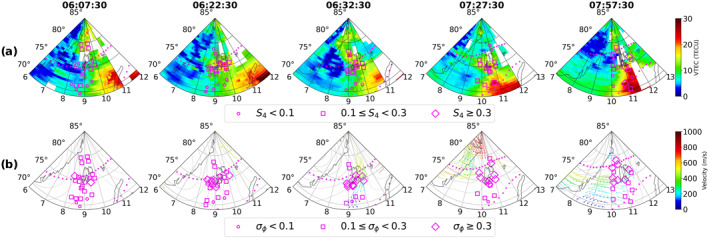
Enhancement in amplitude, phase scintillation inside both the auroral oval and polar cap during interval (B) as multiple high density structures propagated poleward from the sub‐auroral latitudes.

In interval B, $B_{y}$ switches sign multiple times moving the cusp back and forth between prenoon and postnoon sectors (i.e., inside and outside the Svalbard field of view) making interval B the one when Svalbard was most favorably located relative to the cusp auroral region. This interval corresponds to the period when scintillation was simultaneously present in both amplitude and phase of the traversing GNSS radio signals. The TEC maps in Figures 2 and 3 show high density plasma structures in the field‐of‐view of multiple PRNs with the TEC values varying between 20 and 30 TECU in the immediate vicinity of the satellite links. Together with Movies S1 and S2 it is clear that these dense plasma structures originated at sub‐auroral latitudes and then propagated poleward intersecting the raypaths of the PRNs. Figure 4 shows the snapshots of the TEC and SuperDARN maps corresponding to different timestamps during interval (B). Large plasma density enhancements are seen in the eastern half of the field of view (around 11:00 MLT) where the cusp inflow region is located. Significant enhancements in both $S_{4}$ and $\sigma_{\phi }$ were observed both in the auroral oval as well as inside the polar cap as multiple density structures convected across the paths of different GNSS signals into the polar cap. As compared to interval (A), Figure 3b shows that majority of the strongest phase scintillation during interval (B) now occurs poleward at higher latitudes even though dense plasma was present also at lower latitudes near the estimated equatorward edge of the boundary (from DMSP SSUSI).

In interval (C), we show the global ionospheric response when IMF $B_{y}$ turned negative and $B_{z}$ turned northward (see subsection 4.1 for precise IMF values). Since $B_{y}$ is strongly negative, the cusp is shifted toward the prenoon MLT bringing the interval further into the late afternoon local time. As seen in Figure 2a, a depleted region of low density with TEC values ranging between 3 and 10 TECU formed in the polar ionosphere. A closer look in Figure 3a reveals that the intensity of both $S_{4}$ and $\sigma_{\phi }$ diminished significantly with $S_{4}$ reduced to values below the threshold of 0.1 whereas $\sigma_{\phi }$ became very weak. It is worth noting the persistence of both amplitude and phase scintillation at lower latitudes where fast flows and TEC gradients were still observed to exist.

### 4.3 Local Ionospheric Conditions

**Figure 5:**
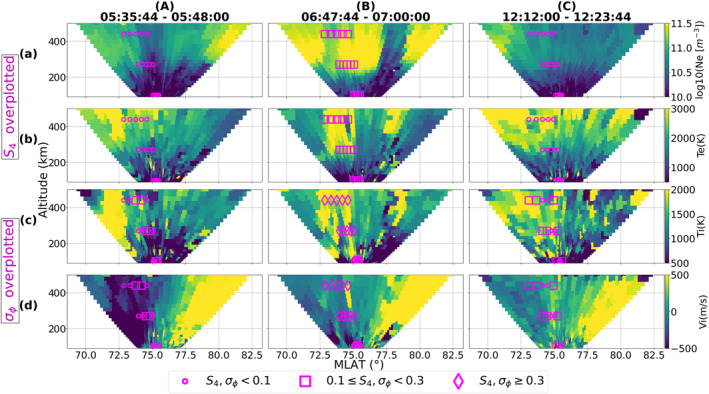
The ESR‐32 m fan plots displaying the altitudinal profiles of $N_{e}$ (panel a), $T_{e}$ (panel b), $T_{i}$ (panel c), $V_{i}$ (panel d). Positive $V_{i}$ values correspond to plasma velocities away from the radar. The fan plots correspond to times within the same three intervals (A), (B), (C) as in Figure 1. Also note that $S_{4}$ indices are overplotted on panels (a), (b) whereas $\sigma_{\phi }$ values are presented on panels (c), (d).

In order to capture the ionospheric conditions of the polar ionosphere during the different intervals of scintillation in detail, we make use of data from the 32 m EISCAT Svalbard radar (ESR‐32 m). As mentioned in Section 2.1, measurements from the ESR‐32 m were collected by operating it under the scanning mode allowing us to infer the ionospheric properties between ∼70°−82° magnetic latitudes during a 12 minute period. Figure 5 shows the ESR‐32 m scan plots for times within the same three different intervals as in Figure 1, that is, before (interval A), during (interval B) and after (interval C) the intense period of simultaneous amplitude and phase scintillation.

During interval (A), the ESR was probing the polar ionosphere with low VTEC but enhanced flow conditions. The average F region electron density in the radar field‐of‐view and within the conjunction zone was $∼6.5\times 10^{10}\text{e}\text{l}/{\text{m}}^{3}$. As seen in Figures 5a and 5b amplitude scintillation was absent whereas Figures 5c and 5d show weak phase scintillation near regions of enhanced $T_{i}$ and $T_{e}$. A channel of enhanced ion velocity directed toward the radar was present close to the region of enhanced $T_{i}$. The associated ion velocity exceeded 500 m/s in agreement with the SuperDARN flow velocities. Notably, a region of fast flow was also observed poleward of the radar without much enhancement in $T_{i}$ and outside the conjunction zone of GNSS signals.

Interval (B) corresponds to the period when intense amplitude and phase scintillation were observed in multiple GNSS signals. Regions of enhanced densities were observed both equatorward and poleward of the radar's field of view with GNSS conjunction occurring only at equatorward latitudes. It is clear from Figure 5a that, during this interval, both $S_{4}$ and $\sigma_{\phi }$ were enhanced in regions of significant plasma densities with the average F region density equaling $∼3.2\times 10^{11}\text{e}\text{l}/{\text{m}}^{3}$. Furthermore, enhanced scintillation was co‐located with narrow regions of enhanced $T_{e}$ as well $T_{i}$ as seen in Figures 5b and 5c. The line‐of‐sight ion velocity was low inside the conjunction zone but was enhanced at latitudes poleward of the radar.

In interval (C), a decrease in the electron density was observed with an average value of $∼7\times 10^{10}\text{e}\text{l}/{\text{m}}^{3}$ within the conjunction zone at F region altitudes. Even though the ionosphere was still active with enhanced levels of $T_{e},T_{i}$, panels (a), (b) show the absence of amplitude scintillation whereas weak phase scintillation persisted during this interval as seen in panels (c), (d).

## 5 Discussion

Combining observations from the set of instruments discussed, the previous section showed the different stages of scintillation development and the associated ionospheric conditions in the polar ionosphere during non‐storm conditions. Three different intervals were chosen to showcase the evolution of scintillation from a period of low activity to an intense state followed by the gradual fading in the intensity back to background levels.

### 5.1 Interval (A)

During interval (A), IMF $B_{z}$ was southward for an extended period of time resulting in the equatorward expansion of the convection cells and auroral boundaries (Cowley & Lockwood, 1992). The corresponding $B_{y}$ component remained mostly positive with the convection cells tilted toward the pre‐noon pre‐midnight direction (see Figure 1a for the $B_{y}$ values and Figure 2b for the convection pattern). This orientation of the cells during positive $B_{y}$ conditions shifts the throat/inflow region (i.e., the entry point of plasma into the polar cap) toward earlier MLTs and is consistent with the findings in Ruohoniemi and Greenwald (1996), Jin et al. (2015). As seen in Figures 2 and 3, signals from multiple GNSS satellites were in regions of enhanced velocities associated with both the sunward return flow as well as the anti‐sunward poleward flow channels even though the associated TEC values were very low. For a time series evolution of the TEC and flow patterns see the Movies S1 and S2. As seen in Figures 1e, 1f, and B1, although there were some moderate bursts in $\sigma_{\phi }$, the average phase scintillation activity during this interval remained weak whereas amplitude scintillation remained absent or weak majority of the times. The ESR‐32 m scans in Figure 5 as well in Movie S3 show an active ionosphere especially with enhanced $T_{i}$ and fast flows. Enhancements in $T_{i}$ in regions of enhanced velocities can be attributed to Joule heating of the ions due to its collision with neutrals (e.g., St.‐Maurice & Hanson, 1982; Skjæveland et al., 2017). The absence of enhanced $S_{4}$ can then be attributed to the lack of enhanced densities or sufficient TEC in the vicinity of the GNSS radio signals. Weak phase without amplitude scintillation then implies that variations in the signal phase were refractive in origin caused by rapid convection of plasma irregularities across the field of view of the GNSS signals (e.g., McCaffrey and Jayachandran (2019), Madhanakumar et al. (2022)).

### 5.2 Interval (B)

In interval (B), the TEC maps in Figures 2, 3, 4 as well as Movies S1 and S2 revealed the presence of large density structures at sub‐auroral latitudes which often developed into patches as a result of detachment from the tongue of ionization (TOI). These structures had the same density as the subauroral plasma with enhancements at least twice the background values and propagated poleward in the anti‐sunward direction. J. Moen et al. (2006) had also observed similar high density plasma structures at sub‐auroral latitudes propagating toward the cusp inflow region using the EISCAT VHF radar. Depending on their location, they are called polar cap patches or blobs if found within or outside the polar cap respectively (Basu et al., 1990; Crowley et al., 2000). As we are only interested on the effects of these dense plasma structures on GNSS signals and not on the distinction between patches or blobs, we would collectively refer to these large density structures as patches for simplicity. Even though many mechanisms have been proposed to explain the formation of patches from high density reservoirs such as solar‐EUV produced plasma and Storm Enhanced Densities (SED), it is beyond the scope of this work to investigate the exact structuring mechanism of the observed patches. Readers interested in the different formation mechanisms are referred to Sojka et al. (1993), Milan et al. (2002), Zhang et al. (2011), Carlson et al. (2006) and references therein. During this interval, Figures 4 and 5 show the poleward propagation of a series of patches with velocities varying between 400 and 700 m/s and which intersected regions of active soft precipitation (visible as enhanced $T_{e}$ at F region altitudes) and enhanced flows/Joule heating (see also Movie S3). Intense bursts in both amplitude and phase scintillation were observed on multiple GNSS links in both the auroral oval and polar cap implying the simultaneous existence of both Fresnel and large‐scale sized irregularities. Assuming a satellite velocity of ∼ 20 m/s and that the relative drift occurs along the horizontal direction, similar to Forte and Radicella (2002), together with an average irregularity drift of 550 m/s obtained from SuperDARN, the range of irregularity scale‐sizes varied between ∼ 50 m ‐ 3 km during periods of scintillation. Jin et al. (2017) observed strongest GPS phase scintillation associated with the combination of polar cap patches and auroral dynamics, followed by moderate scintillation in the case of cusp dynamics without polar cap patches, and weak scintillation with patches outside the cusp aurora. In this study, we also observed the phase scintillation to be the strongest in the oval whereas it remained moderate inside the polar cap majority of the times. Moreover, the strength of amplitude scintillation remained in the weak‐moderate regime in both the auroral oval as well as inside the polar cap suggesting that the convecting dense plasma maintained the small‐scale structuring irrespective of whether the patches were in regions of auroral dynamics or not. Thayyil et al. (2021) had similarly observed patches maintaining the integrity of Fresnel structures in the leading edge as they convected across nightside the polar cap. The Movie S3 shows some events where the 1‐min $S_{4}$ index was below 0.1 even though dense plasma was present in regions of enhanced $T_{e}$ and $T_{i}$. However, the 1‐s $S_{4}$ was enhanced and remained above the threshold of 0.1 for these events. This discrepancy between the 1‐s and 1‐min indices could be because of the fact that the scintillation events were short lived and the 1‐min index missed these intense bursts as a result of averaging over a larger window. The enhancement in the 1‐s $S_{4}$ index then suggests that Fresnel sized irregularities were in fact present during such events. At the nightside sector, Nishimura et al. (2023) had similarly observed the 1‐s $S_{4}$ index to be a better proxy than the 1‐min index to capture the intense and short‐lived bursts in amplitude scintillation associated with various auroral forms. Jiao et al. (2013) had observed amplitude scintillation events to be brief and lasting only for 3.7 min in duration. Even though small‐scale irregularities have shorter life‐span and dissipate faster, we however observed amplitude scintillation (and hence small‐scale irregularities) to persist for more than 1 hour in duration.

**Figure 6:**
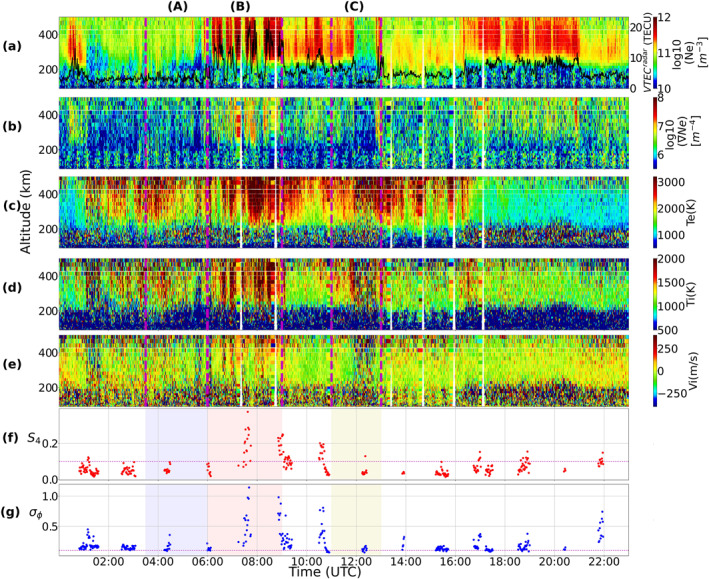
The field‐aligned measurements from the ESR‐42 m displaying the height profiles of $N_{e}$ (panel a), gradients in density $\left(\nabla N_{e}\right)$ in panel (b), $T_{e}$ (panel c), $T_{i}$ (panel d) and $V_{i}$ (panel e). Panels (f), (g) contain the $S_{4},\sigma_{\phi }$ values when conjunctions were present between the ESR‐42 m beams and GNSS radio signals. The three intervals are separated using dashed vertical magenta lines in panels (a–e) and shaded in panels (f–g).

In order to complement the observations made by the ESR‐32 m, we also used the measurements from the field‐aligned ESR‐42 m. Figure 6 shows the measurements collected by the ESR‐42 m for the same day and covering the same three intervals (A), (B), (C). Together with the usual ionospheric parameters $N_{e},T_{e},T_{i},V_{i}$, panel (b) shows the gradients in electron density $\left(\nabla N_{e}\right)$ whereas the vertical TEC from radar density measurements $\left(VTEC^{\mathrm{radar}}\right)$ is plotted on panel (a) alongside $N_{e}$. The gradients were calculated from the electron density measurements using Equation 2 and following the procedure described in the methodology section. There is increased noise below 200 km due to a technical problem which however does not affect our results or discussions. Panels (f) and (g) contain the scintillation indices, $S_{4}$ and $\sigma_{\phi }$, which are only plotted during times when there were conjunctions between the radar and GNSS beams. On comparing intervals (A), (B), (C) we clearly observe gradients associated with large density structures that are co‐located with regions of enhanced $T_{e}$ and $T_{i}$ during interval (B). Elevated electron temperatures near regions of enhanced densities at F region altitudes indicate ongoing soft particle precipitation (Doe et al., 2001; Kersley et al., 1988; Vontrat‐Reberac et al., 2001) whereas enhanced $T_{i}$ indicate Joule heating of the ions (St.‐Maurice & Hanson, 1982). The $S_{4}$ and $\sigma_{\phi }$ values are significantly enhanced from the background level during this interval. Together with Figure 5 this confirms the presence of irregularities from Fresnel to large‐scales with sufficient strengths to perturb both the amplitude and phase of GNSS signals. These results are in line with the observations in Mitchell et al. (2005), Kintner et al. (2007), Jenner et al. (2020), Madhanakumar et al. (2024) where the authors associated scintillation occurrence to the presence of gradients in density structures. Notably, the vertical TEC observed by the radar closely matched the GNSS VTEC values observed in Figures 2 and 3.

A necessary condition for the production of irregularities is the existence of sources of free energy. Multiple mechanisms can then structure the plasma to produce scintillation causing irregularities in the high latitude ionosphere. Sheared plasma flow perpendicular to magnetic field can give rise to Kelvin‐Helmholtz instability (KHI), generating medium and large‐scale irregularities (Basu et al., 1988; Keskinen et al., 1988; Oksavik et al., 2011). An increased plasma flow in the direction of density gradients can then efficiently generate smaller scale irregularities through Gradient‐Drift instability (GDI) (Burston et al., 2009; Carlson et al., 2007, 2008; Tsunoda, 1988; Weber et al., 1984) such as in the trailing edges of polar cap patches. Spicher et al. (2020) had shown KHI to be an effective mechanism in generating density irregularities within minutes. GDI could then efficiently operate on these intermediate scale structures generating small‐scale irregularities within tens of seconds (Carlson et al., 2007; Oksavik et al., 2012; J. Moen et al., 2012; Spicher et al., 2015). In addition to KHI, particle precipitation is also suggested to be the source of large‐scale irregularities which can be further structured down to small‐scales by GDI (Goodwin et al., 2015; Kelley et al., 1982; Oksavik et al., 2006, 2012). Roble and Rees (1977), Valladares et al. (1994) have shown that density variations of the order of 20%−30% in the F region can be achieved within 15–20 min. Assuming a production rate of ionization at F region altitude to be $∼8\times 10^{8}\;{\text{m}}^{-3}{\text{s}}^{-1}$ from Millward et al. (1999)'s precipitation model, Hosokawa et al. (2016) estimated that the electron density in poleward moving auroral forms (PMAFs) increases by $2-3\times 10^{11}\;{\text{m}}^{-3}$ within 5 min. Given a high density structure such as polar cap patches, KHI or soft precipitation can therefore structure the dense plasma producing large to intermediate scale irregularities within minutes which can then cascade into Fresnel scale structures that can cause simultaneous amplitude and phase scintillation in GNSS signals. Ivarsen et al. (2023), on the other hand, had found that the energy flux of precipitating particles decreases or remains unchanged with increasing geomagnetic activity whereas ionospheric irregularities and the associated phase scintillation increases significantly. The authors therefore suggested that other sources played a major role in producing irregularities associated with GNSS phase scintillation rather than soft precipitation during disturbed conditions.

We now proceed to place our scintillation observations on the $7^{th}$ of November 2013 within the context of the aforementioned different mechanisms/processes. Referring back to interval (B) in Figures 2, 3, 4, we observed scintillation (predominantly in phase) even at lower latitudes as plasma structures of enhanced density entered the auroral oval. Furthermore, scintillation was present along the sides and inside these density enhancements indicating that they were already structured and had irregularities throughout. Similar observations of the presence of irregularities at both the edges and/or throughout regions of enhanced plasma density such as polar cap patches were also made by Gondarenko et al. (2003), Gondarenko and Guzdar (2004), Coker et al. (2004), Thayyil et al. (2021) and references therein. Interval (B) in Figures 5 and 6 show scintillation to be associated with dense plasma structures and in regions of enhanced $T_{i}$ (or fast flows). This can provide the necessary conditions for KHI to evolve and grow. Similar to Carlson et al. (2007), the growth time of KHI was estimated using the maximized growth time equation:

(3) $$\tau_{\mathrm{KHI}}=\frac{5L}{V}$$

Using a velocity difference (V) of 300 m/s (from SuperDARN) and assuming a velocity difference scale length (L) of 10 km, similar to Carlson et al. (2007), the growth time of KHI was estimated to be about 3 min. In addition, ongoing soft particle precipitation was also observed during this interval. With an average convection velocity ranging between 400 and 700 m/s, as measured by SuperDARN, and assuming a latitudinal width of 3° for the auroral oval it would take approximately 7–10 min for the patches to transit across the auroral oval to the polar cap. If we assume an F region ionization production rate of $∼8\times 10^{8}\;{\text{m}}^{-3}{\text{s}}^{-1}$ from Millward et al. (1999), an estimated density increase of the order of $1.5-2.5\times 10^{11}\;\text{e}\text{l}/{\text{m}}^{3}$ can occur within 3–5 min. This suggests that plasma pre‐structuring can be achieved by soft precipitation within minutes when patches are inside the auroral oval. With a larger precipitating flux, the plasma structuring can occur even faster. Furthermore, the presence of strong gradients associated with the density structures, as seen in Figure 6b, suggests that GDI could be active during this interval. The growth time for GDI in the linear regime can be calculated when k⋅B=0, as (Tsunoda, 1988):

(4) $$\tau_{\mathrm{GDI}}=\frac{L}{v_{0}}$$

with the gradient length scale, L, given as:

(5) $$L=\frac{N_{0}}{\Delta N_{e}/\Delta x}$$

Here, $v_{0}$ is the relative velocity of the plasma with respect to the neutrals, $\Delta N_{e}/\Delta x$ the density gradients and $N_{0}$ the background density. Using gradient and background density information from Figure (6) along with the plasma velocity from SuperDARN, GDI was estimated to grow as fast as 35 s using Equation 4. For the calculation, velocity of the neutral gas was ignored similar to J. Moen et al. (2012), Oksavik et al. (2012), Spicher et al. (2015).

We now proceed to provide a likely scenario of irregularity generation based on our observations of scintillation and the above discussed sources/instability mechanisms. Figures 2, 3, 4, 5, 6 reveal that the strongest scintillation occurred when large densities and gradients were present in regions of strong flows and particle precipitation. This suggests that TOIs and (or) polar cap patches provided the necessary large background density with KHI and soft particle precipitation structuring the plasma as the patches convected poleward. The enhancement in the phase scintillation could then be associated with the generation of large to intermediate scale sized irregularities within minutes as a result of fast flows and hence KHI as in Spicher et al. (2020) or to irregularity structuring by particle precipitation as in Kinrade et al. (2012), Makarevich et al. (2021), Oksavik et al. (2015). GDI could have further operated to produce the small‐scale irregularities within seconds resulting in simultaneous amplitude scintillation in GNSS frequencies. It is worth pointing out that we are only able to provide estimates for the growth times of the different processes/instabilities due to our assumptions and uncertainties associated with different parameters used in Equations (3), (4), (5). Nevertheless, the estimates suggest that our observations of intense amplitude and phase scintillation, and hence the presence of irregularities of varying scale‐sizes, are consistent with different irregularity generation mechanisms/processes. We however emphasize the need to conduct more studies to understand the effectiveness of different sources/instability mechanisms and their relative importance in generating a spectrum of irregularities that can simultaneously affect both the amplitude and phase of GNSS signals in the polar ionosphere.

### 5.3 Interval (C)

During interval (C), the ESR‐32 m field‐of‐view in the post‐noon sector was covered by ionospheric plasma of depleted density as compared to interval (B) and similar to interval (A). Below we briefly discuss the formation process of this depletion region that led to the significant removal of scintillation in GNSS signals. Referring to Figure 1a, this interval corresponds to the period when IMF $B_{y}$ turned from being positive to strongly negative whereas $B_{z}$ turned northward after being strongly southward. A closer look at the time history of the TEC maps (see Movie S1) during this interval shows a TOI being cut to form a patch that propagated poleward just before the depletion region covered the polar cap ionosphere. The detachment was similar to the one observed in Milan et al. (2002) and occurred when the plasma flow alternated between poleward and zonal directions near the cusp region as a result of $B_{y}$ reversal, changing the entry point of the plasma into the high latitude polar cap (Rodger et al., 1994). Valladares et al. (1994) observed fast plasma jets with velocities exceeding 2,000 m/s and $T_{i}$ in excess of 5,000 K in the pre‐noon sector during IMF $B_{y}$ negative conditions. As the region of plasma jets were co‐located with regions of low F region densities, the authors suggested the enhanced recombination rate of $O^{+}+N_{2}$ reaction as the decisive factor that led to the depletion in the electron density. However, in our study the ion velocities, as measured by SuperDARN, rarely exceeded 700 m/s during interval (C). Furthermore, comparing the ESR‐32 m scans of $T_{i}$ in Figure 5c during intervals (B) and (C), we observe similar regions of ion frictional heating but density was severely depleted only during interval (C) suggesting that Joule heating alone cannot account for the significant density depletion observed across the radar field‐of‐view in line with the observations in Milan et al. (2002). Similar conclusions were reached by Ren et al. (2020) as well after the authors compared their observed density depletion in the Sondrestrom ISR to the estimated decrease due to Joule heating. The authors suggested that ion frictional heating could not account for the observed depletion region above 300 km in the ISR scans as well as in their TEC maps. As an alternate solution, the transport of low density structures from the dawn or dusk sector, depending on the sign of IMF $B_{y}$, was offered to explain the formation of the density depletion in the cusp/polar cap regions (Milan et al., 2002; Ren et al., 2020). In fact, the time evolution of TEC across the polar ionosphere between 11:00 ‐ 13:00 UTC (see Movie S1) revealed a depleted region of TEC being drawn into the post‐noon cusp/polar cap from the westward‐directed return flow channel in the dawn sector during negative $B_{y}$ conditions. We therefore conclude that the transport of this low density plasma played a dominant role in the erosion of irregularities in the post‐noon sector leading to the significant weakening of amplitude and phase scintillation in GNSS signals.

To summarize, a comparison of scintillation strengths during intervals (B) and (C) in Figures 1e and 1f revealed that the most intense period of scintillation occurred during interval (B), that is, in the pre‐noon sector. Observations from the ESR (both 32 and 42 m) in Figures 5 and 6 show that the most striking differences between the two intervals are in the electron densities and gradients. Even though both electron and ion temperatures during interval (C) appear to be noisy, due to low density for radar back‐scatter, enhanced regions of $T_{e}$ and $T_{i}$ were still present though with reduced intensity as compared to interval (B). Together with Figure 6a, which shows a depleted region of ionospheric plasma with low $VTEC^{\mathrm{radar}}$ between 12:00 ‐ 13:00 UTC, Figure 6b shows that the ionosphere, during interval (C), was devoid of significant density gradients when compared to interval (B). The notable reduction in the strength of amplitude scintillation during interval (C) then suggests that fast flows (resulting in enhanced $T_{i}$) and ongoing soft precipitation become less effective in creating Fresnel sized irregularities when regions of dense plasma are absent.

## 6 Conclusion

We have presented a multiscale study of GNSS scintillation in the dayside polar ionosphere using a suite of both ground‐based and space borne instruments during non‐storm conditions. The different evolutionary stages of both amplitude and phase scintillation, from a state of low disturbance to intensification followed by the reduction in strength, were investigated in detail and the observations were placed in relation to the changes in the IMF $B_{y},B_{z}$ components. Additionally, attempts were made to understand the role of different instability mechanisms in the high latitude ionosphere that could help generate irregularities of various scale‐sizes capable of inducing simultaneous amplitude and phase scintillation in GNSS signals. The key observations and interpretations of this study are summarized below.

1. Intense amplitude and phase scintillation, lasting for ∼1 hour, were observed in the pre‐noon sector during non‐storm conditions with the 1‐s $S_{4}$ reaching a maximum value of 0.75 whereas $\sigma_{\phi }$ reached values up to 2.2 rad.
2. Fresnel scale irregularities were observed both in the auroral oval as well as inside the polar cap with the source being high density structures transported from subauroral latitudes. Associated amplitude scintillation in both the regions exhibited similar strengths suggesting that Fresnel structures maintained significant power regardless of being located in regions with auroral dynamics or not.
3. The convecting dense structures intersected regions of strong ion heating (i.e., enhanced flows) and structured soft particle precipitation, and were observed to be associated with significant density gradients.
4. The northward turning of the IMF $B_{z}$ component together with the strong negative excursion of the $B_{y}$ component resulted in the westward transport of a region of depleted density into the post‐noon sector from the dawn sector. This led to the erosion of Fresnel and large‐scale sized irregularities resulting in the significant weakening of amplitude and phase scintillation in the post‐noon sector.

Our observations and results complement the existing literature where intense amplitude scintillation were frequently observed to occur during severe geomagnetic conditions and in the post‐noon sector, with large densities playing a crucial role in the occurrence and strength of scintillation in GNSS signals. The findings presented here suggest that geomagnetic conditions cannot always capture the true ionospheric response on radio signals such as that from GNSS, making scintillation forecasting solely based on geomagnetic activity difficult. Furthermore, we emphasize that the plasma intake from lower latitudes in the pre‐noon sector can be equally important as that from the post‐noon sector for scintillation studies. In addition, even though post‐noon sector is an excellent breeding ground for intense irregularities of different scale‐sizes that can significantly perturb GNSS signals, the IMF $B_{y}$ and $B_{z}$ components can help in the transport of low density plasma thereby removing/significantly reducing scintillation. Further work has to be done to reveal how often the reversals in IMF components during non‐storm conditions lead to plasma depletion in the post‐noon sector and its subsequent effect on scintillation in GNSS signals.

## Supporting information

Supporting Information S1

Movie S1

Movie S2

Movie S3

## Data Availability

Data from EISCAT can be obtained from the Madrigal database (Häggström, 2013). The GNSS data from KHO, NYA and BJN can be obtained from the University of Bergen Global Navigation Satellite System Data Collection (Oksavik, 2020b). The TEC data used in TEC maps are accessible from the Madrigal database (Coster, 2013). Raw SuperDARN data with DOI's can be accessed via SuperDARN data collection (SuperDARN, 2021). Processing to higher level products is done using the Radar Software Toolkit (SuperDARN Data Analysis Working Group, 2022). Data for the IMF components, solar wind, SYMH and the auroral electroject indices can be obtained from OMNI database (King & Papitashvili, 2013). The auroral boundary data is obtained from the DMSP SSUSI instrument (Paxton, 2013).

## Appendix A Overview of Instruments/Data Sources

**Table A1:** Overview of the Instruments/Data Sources, Measurement Quantities As Well As Their Coverage Used in the Study

Data source/instrument	Latitude	Longitude	Measurement
EISCAT 32 m (ESR‐32 m)	78.15°	16.02°	$N_{e},T_{e},T_{i},V_{i}$
EISCAT 42 m (ESR‐42 m)	78.15°	16.02°	$N_{e},T_{e},T_{i},V_{i}$
NovAtel GPStation‐6 GISTM	78.1° (KHO), 74.5° (BJN), 78.9° (NYA)	16.0° (KHO), 19.0° (BJN), 11.9° (NYA)	$S_{4},\sigma_{\phi },VTEC,ROT$
CEDAR Madrigal	60°−90°	0°−360°	VTEC
SuperDARN	60°−90°	0°−360°	Convection velocity, electric potential
DMSP SSUSI	60°−90°	0°−360°	Poleward, equatorward auroral boundaries
OMNI	N/A	N/A	$B_{x},B_{y},B_{z}$, flow speed, pressure, proton density
World Data Center for Geomagnetism	(−34°)−48°	2°−293°	SYMH
World Data Center for Geomagnetism	56.5°−78°	(−157°)−171°	AL, AU, AE
