# Supplementary material for: The Growth and Decay of Intense GNSS Amplitude and Phase Scintillation During Non‐Storm Conditions
Source: Space Weather. 2024 Nov 30;22(12):e2024SW004108. doi: 10.1029/2024SW004108 (PMC11607637; doi:10.1029/2024SW004108)
Supplement: Supplementary file 1 — Supporting Information S1 [file SWE-22-0-s001.pdf]

AGU PUBLICATIONS

Space Weather Journal

Supporting Information for

The growth and decay of intense GNSS amplitude and phase scintillation during non-storm conditions

Mahith Madhanakumar[1], Andres Spicher[1], Juha Vierinen[1], Kjellmar Oksavik[2,3], Anthea J. Coster[4], Devin Ray Huyghebaert[1,5], Carley J. Martin[5], Ingemar Häggström[6], Larry J. Paxton[7]

[1]Department of Physics and Technology, UiT The Arctic University of Norway, Tromsø, Norway

[2]Department of Physics and Technology, University of Bergen, Norway

[3]Arctic Geophysics, University Centre in Svalbard, Longyearbyen, Norway

[4]Haystack Observatory, Massachusetts Institute of Technology, Westford, MA, USA

[5]Institute of Space and Atmospheric Studies, University of Saskatchewan

[6]EISCAT Scientific Association, Kiruna, Sweden

[7]The Johns Hopkins University Applied Physics Laboratory, Laurel, MD, USA

Contents of this file

Captions for Movies S1 to S3

Additional Supporting Information (Files uploaded separately)

Movies S1, S2 and S3.

Introduction

This supporting information provides the captions for Movies S1, S2 and S3 that have been referred to in the manuscript. Movie S1 shows the global evolution of TEC and SuperDARN convection velocities, and supports Figure 2 of the manuscript. Movie S2 shows the TEC and SuperDARN maps above Svalbard and supports Figures 3 and 4. Movie S3 corresponds to the EISCAT-32m (ESR-32m) scan plots and supports Figure 5.

Movie Captions

Movie S1. Global TEC and SuperDARN convection velocity maps between 03:30 – 13:00 UTC (i.e. spanning the 3 different intervals (A), (B), (C)). S_4 indices are overplotted on the TEC maps whereas σ_ϕ values are overlaid on the SuperDARN maps. Legends are shown at the bottom. Poleward and equatorward auroral boundaries from DMSP SSUSI are also plotted on both the maps. The IMF By, Bz components are shown on the top-right corner of the maps.

The transport of a region of depleted density from the dawn sector into the post-noon sector of the polar ionosphere can be clearly seen between 11:30 – 12:30 UTC which resulted in the significant weakening of both amplitude and phase scintillation in the polar ionosphere.

Movie S2. TEC and SuperDARN convection velocity maps above Svalbard archipelago between 03:30 – 13:00 UTC in the same format as **Movie S1.**

The poleward transport of patches as a result of detachment from the tongue of ionisation (TOI) from sub-auroral latitudes can be seen starting from 06:00 UTC. Bursts in amplitude scintillation in both the auroral oval and polar cap can be seen

(simultaneously with phase scintillation) as patches moved across the field-of-view of different GNSS PRNs.

Movie S3. ESR-32m scan plots between 03:30 – 13:00 UTC. Similar to Figure 5, S_4 is overplotted on N_e and T_e panels whereas σ_ϕ values are overlaid on T_i and V_i panels.

.
